# Magnetic resonance imaging for brain stereotactic radiotherapy: A review of requirements and pitfalls

**DOI:** 10.1007/s00066-020-01604-0

**Published:** 2020-03-23

**Authors:** Florian Putz, Veit Mengling, Rosalind Perrin, Siti Masitho, Thomas Weissmann, Johannes Rösch, Tobias Bäuerle, Rolf Janka, Alexander Cavallaro, Michael Uder, Patrick Amarteifio, Sylvain Doussin, Manuel Alexander Schmidt, Arndt Dörfler, Sabine Semrau, Sebastian Lettmaier, Rainer Fietkau, Christoph Bert

**Affiliations:** 1 Department of Radiation Oncology, Friedrich-Alexander-University Erlangen-Nürnberg, Universitätsstraße 27, 91054 Erlangen, Germany; 2 Institute of Radiology, Friedrich-Alexander-University Erlangen-Nürnberg, Ulmenweg 18, 91054 Erlangen, Germany; 3 Imaging Science Institute Erlangen, Siemens Healthineers, Ulmenweg 18, 91054 Erlangen, Germany; 4 Department of Neuroradiology, Friedrich-Alexander-University Erlangen-Nürnberg, Schwabachanlage 6, 91054 Erlangen, Germany

**Keywords:** Radiosurgery, Distortion correction, Local control, Radiotherapy simulation, Radiotherapy treatment planning

## Abstract

Due to its superior soft tissue contrast, magnetic resonance imaging (MRI) is essential for many radiotherapy treatment indications. This is especially true for treatment planning in intracranial tumors, where MRI has a long-standing history for target delineation in clinical practice. Despite its routine use, care has to be taken when selecting and acquiring MRI studies for the purpose of radiotherapy treatment planning. Requirements on MRI are particularly demanding for intracranial stereotactic radiotherapy, where accurate imaging has a critical role in treatment success. However, MR images acquired for routine radiological assessment are frequently unsuitable for high-precision stereotactic radiotherapy as the requirements for imaging are significantly different for radiotherapy planning and diagnostic radiology. To assure that optimal imaging is used for treatment planning, the radiation oncologist needs proper knowledge of the most important requirements concerning the use of MRI in brain stereotactic radiotherapy. In the present review, we summarize and discuss the most relevant issues when using MR images for target volume delineation in intracranial stereotactic radiotherapy.

## Introduction

Magnetic resonance imaging (MRI) was introduced many decades ago for target volume delineation in brain tumors [1–4]. The far superior depiction of intracranial malignancies and organs at risk (OAR) has been the basis for the unrivaled success of MRI in intracranial treatment planning. It is because of this long-standing history and the fact that nowadays MRI is used in nearly every patient with an intracranial tumor, that the radio-oncologist as a result of a false sense of security stemming from the perceived familiarity with its use may be unaware of the potential dangers and pitfalls associated with using MRI in an indiscriminate way in daily clinical practice. In fact, the relationship between MRI and radiotherapy is more complicated than daily routine would suggest. Distortions present in MR images are a good example of a source of treatment error that overshadowed the introduction of MRI in radiotherapy [2–4] but may still endanger treatment success in high-precision stereotactic radiosurgery of brain metastases today [5, 6]. The main reason why some of the perils associated with MRI-based radiotherapy treatment planning have still not completely disappeared may be the fact that MRI for the most part has been outside the scope of the radio-oncologist. While the planning CT is usually located in the radiotherapy treatment facility and is optimized for the requirements of radiotherapy, MRI studies for radiotherapy treatment planning are frequently performed in external departments. However, the diagnostic radiologist who acquires these MR studies may be less familiar with the specific requirements of radiotherapy treatment planning, as the requirements on imaging for radiotherapy and diagnostic radiology are significantly different. Therefore, the radiation oncologist needs proper knowledge of the most important caveats and considerations concerning the use of MRI in brain stereotactic radiotherapy.

For stereotactic radiotherapy very high and specific technological quality requirements are needed to assure highly precise treatment delivery. These requirements have been comprehensively formulated and defined in the accompanying DEGRO/DGMP editorial and the DGMP review in the present issue [7, 8]. Optimal MR imaging is a crucial element for high overall accuracy in stereotactic radiotherapy and ultimately a necessity for treatment success but is frequently overshadowed by the technological requirements that are more closely related to treatment delivery. In light of commonly used gross target volume (GTV) to planning target volume (PTV) margins of ≤1 mm, the requirements for MR imaging are particularly demanding in intracranial stereotactic radiotherapy [9, 10].

Accompanying the very important official articles of the DEGRO and DGMP working groups, in the present review, we therefore highlight the important role of optimal MR imaging by summarizing and discussing the most relevant issues arising when using MRI in treatment planning for brain stereotactic radiotherapy.

## Time between MR imaging and treatment delivery

**Fig. 1:**
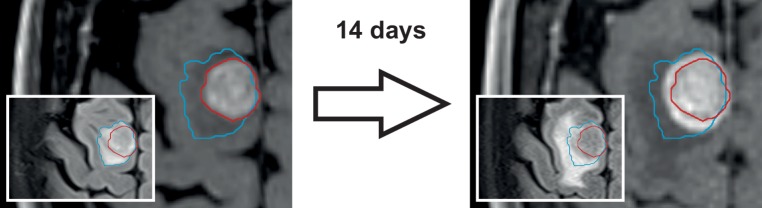
Importance of the time interval between MR imaging and treatment delivery. Example of growth in a melanoma brain metastasis that would have resulted in geographic miss. The 1.5 cm-diameter tumor increased from 2.1 to 3.0 cm^3^ during a time interval of 14 days (postcontrast T1-MPRAGE). *Note also:* Substantial increase in perifocal edema (inset: T2-FLAIR)

One of the most crucial parameters for treatment precision is the interval between MR imaging and treatment delivery [11, 12]. This is especially important for brain metastases that have a high rate of growth [12, 13] and are frequently surrounded by fluctuating amounts of perifocal edema [14], which may undergo profound changes spontaneously or when corticosteroid dosage is modified (Fig. 1; [15]).

Importantly, Seymour et al. found worse local control for brain metastases if the interval between MR imaging and stereotactic radiosurgery was ≥14 days (local control 56% vs. 95% at 6 months post-SRS) [11]. Salkeld et al. even found profound changes with imaging intervals ≤7 days before SRS. Change in management was required for 41% of patients with interval ≤7 days and even for 78% if the delay exceeded 7 days. The most frequent reason for replanning was an increase in tumor or resection cavity volume [12, 16]. Therefore, the interval between imaging and treatment delivery should be as short as possible. While same-day imaging would be optimal, in our university medical center in Erlangen we have established the requirement that the interval between imaging and treatment delivery must not exceed 5 days.

### Need for repeated MR imaging during radiotherapy

**Fig. 2:**
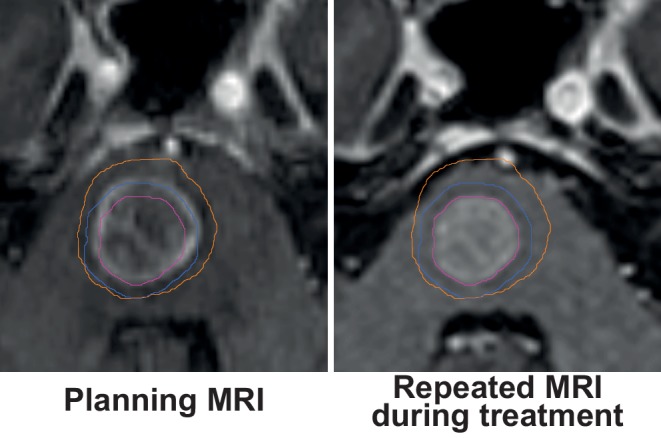
Repeat simulation MRI during fractionated stereotactic radiotherapy in a patient with brainstem metastasis (postcontrast T1-MPRAGE). *Note:* Substantial reduction in tumor volume and in accompanying edema results in profound shifting of the brainstem. The radiotherapy plan was adapted based on the repeated planning MRI

Brain metastases and primary brain tumors undergoing fractionated stereotactic radiotherapy may undergo profound changes during treatment due to transient swelling, changes in perifocal edema and treatment response (Fig. 2). Hessen et al. in a recent study evaluated the significance of a repeated MRI scan in the fractionated stereotactic radiotherapy of 18 brain metastases and 20 resection cavities. For cases with in situ brain metastases, reductions in PTV coverage of up to 34.8% were found. Interestingly, changes were less pronounced for postoperative cases (up to −4.5% in PTV coverage) and pretreatment changes were predictive of reduced coverage during treatment [17]. Importantly, as only 3–5 fractions were employed in the study by Hessen et al., even more pronounced changes would be expected with more prolonged fractionation schemes. At our university medical center in Erlangen we usually repeat imaging during prolonged courses of stereotactic treatment of brain metastases or primary brain tumors at least once, especially when risk factors like large edema, hygroma and other similar pathologies are present.

## Patient positioning for simulation MRI

The simulation MRI for intracranial radiotherapy is most frequently acquired in a diagnostic head coil and subsequently rigidly coregistered to the planning CT obtained in the treatment position with immobilization. However, acquiring the simulation MRI in the treatment position using an immobilization mask similar to the planning CT could decrease errors due to nonrigid tissue deformation, reduce uncertainties related to image registration, reduce motion artifacts and may even serve as the basis for an MR-only workflow using synthetic CT.

**Fig. 3:**
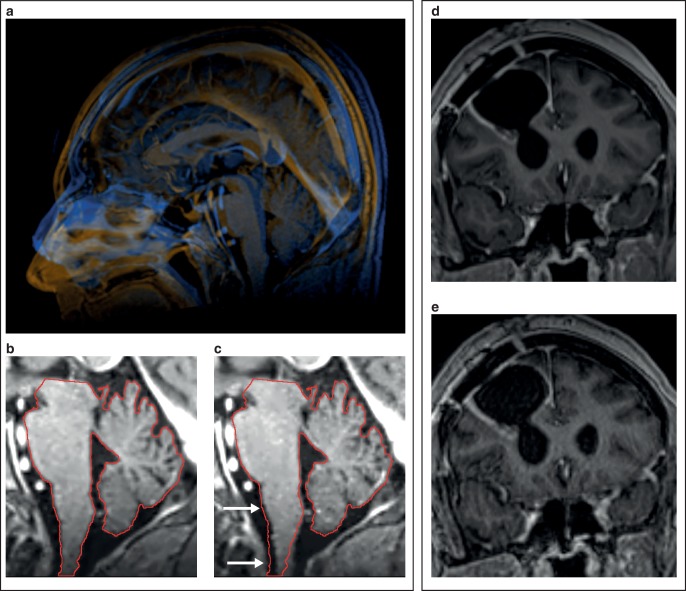
Patient positioning for simulation MRI. **a** Simulation MRI with mask immobilization vs. in a diagnostic head coil. The head is substantially flexed in the diagnostic head coil (*amber*) compared to the simulation MRI with mask immobilization (*blue*) leading to slight displacement of infratentorial structures (**b**, **c**, *white arrows*). **d**, **e** Reduced motion artifacts in the stereotactic mask system (**d**) compared to the diagnostic head coil (**e**)

Deformation and displacement of brain tissues in different imaging positions is certainly more limited than deformation of organs at most extracranial sites [6, 18]. However, patient positioning in a routine radiologic setup as opposed to the treatment position with mask immobilization frequently results in a different head extension [19]. Bending of the brainstem and slight displacement of infratentorial structures may occur due to different angles of extension at the occipito-atlanto-axial joint complex, which could become important when treating targets in the medulla oblongata, caudal pons or the cerebellar vermis (Fig. 3a–c; [20]).

Aside from avoiding slight infratentorial tissue deformation, the accuracy of rigid registration may also improve when acquiring the simulation MRI in the treatment position [6, 21]. In general, uncertainties of ≤0.5 mm along the X‑ and Y‑axis in-plane and of ≤1 mm along the Z‑axis have been reported for normalized-mutual-information based MRI–CT coregistration for a CT slice thickness of 2–3 mm and a 1 × 1 × 2 mm^3^ 3D-T1-MPRAGE sequence. Importantly, when using a CT slice thickness of 5 mm and a 1 × 1 × 5 mm^3^ 2D-SE sequence the registration uncertainty rose by a factor of 2–3 [22]. High MR or CT slice thickness and using 2D MR sequences with gaps could be particularly detrimental to registration accuracy in the presence of differing head extension. In these cases, planes of high in-plane-image resolution of the MRI and planning CT are tilted. It is currently unclear, however, if an MRI acquired with mask immobilization can further improve registration accuracy when the present recommendations for imaging are followed (planning CT with ≤1 mm slice thickness and ≤1 mm^3^ T1 3D-sequence [6]) and proper registration software is used.

Performing MRI studies in the treatment position with mask immobilization, however, decreases patient motion, which may help with precise target volume definition (Fig. 3d–e; [23]).

A few centers have established solutions for acquiring simulation MRIs with mask immobilization [21, 23, 24]. As most stereotactic mask systems will not fit into routine radiologic head coils, most groups have used a flexible coil setup, which could degrade image quality [6, 23, 25]. According to the current consensus imaging with mask immobilization as well as imaging in a routine radiologic setup are both appropriate strategies for MR simulation in intracranial radiotherapy if MR images are registered to a planning CT [6].

### Promises of synthetic CT and MR-only planning

Synthetic CT, which is the calculation of synthetic CT images from one or multiple MR sequences, promises to remove the need for an additional planning CT and thus any uncertainties with MR–CT registration [26]. Solutions by different manufacturers are already available. While eliminating registration uncertainties, the use of synthetic CT may potentially introduce errors into treatment planning, due to bulk assignment of CT numbers to segmented tissues and in some algorithms the use of atlases to generate major bones [26]. Current methods provide reasonable results in most standard situations and dosimetric differences <1% have been reported for certain atlas and voxel-based methods [26]. Importantly, some methods of synthetic CT generation may require nonstandard sequences that increase measurement time [27]. Deep learning-based approaches are at the forefront of current research on synthetic CT and have been shown to outperform other published approaches in terms of mean absolute HU errors in a recent study [28]. These solutions could allow for fast and robust synthetic CT generation from standard MR sequences.

As MR–CT registration has been shown to reduce positional errors introduced by MRI, minimization of MR distortions becomes even more relevant, when opting for an MR-only workflow [29].

## Accounting for distortions in MR images

**Fig. 4:**
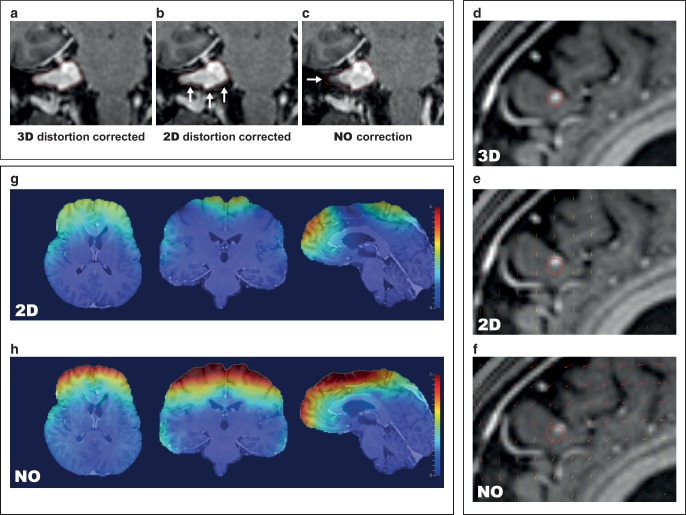
Impact of gradient nonlinearity distortion correction. **a**–**c** Example of a case with vestibular schwannoma. Comparison of vendor-specific 3D- (**a**) and 2D-distortion correction (**b**) with the uncorrected T1-MPRAGE dataset (**c**). Tumor outline (*red*) from the 3D-corrected dataset is projected onto the 2D-corrected and uncorrected dataset for reference. *Note*: While the difference between 2D- and 3D-correction is more subtle, the 2D-corrected dataset underestimates the lower border of the tumor (*white arrows*). **d** Example in a patient with a prefrontal brain metastasis. Sagittal view. The tumor is significantly displaced in the uncorrected (**f**) and 2D-corrected dataset (**e**). *Arrows* indicate the amount of tissue distortion in the 2D- and uncorrected images with reference to the 3D-corrected dataset. *Note*: The 2D-corrected dataset contains residual through-plane distortions. **g**, **h** Amount of displacement with reference to 3D-corrected images overlaid as heatmap. *Blue* indicates no displacement, *red* indicates a displacement of 2 mm. While 2D-corrected images (**g**) show fewer residual distortions than uncorrected images (**h**), distortions of up to 2 mm were still present after 2D-correction. *Note*: The amount of gradient nonlinearity-related distortions for other scanner models and patient positions is different from the examples shown here. Additional sources of distortion include magnet imperfections and patient-induced susceptibility-related distortions, which are not visualized here

While CT scans can be considered geometrically accurate, several mechanisms may lead to distortions in MR images that endanger precise treatment delivery [2, 3, 30, 31]. Slight distortions on the order of 1–2 mm are nearly impossible to identify in MR images, even when coregistering to a planning CT. In general, distortions in MR images are nonlinear and unevenly distributed across the image dataset. Image distortions are usually most pronounced at the periphery and least problematic near the isocenter of the MR scanner (Fig. 4g–h; [4, 32–34]). In clinical practice most distortions will therefore be expected to occur at the periphery of the brain especially near air–bone interfaces, which translates among others to the frontopolar and orbitofrontal cortex but also to cranial aspects of the prefrontal cortex and to lateral and inferior parts of the temporal lobe.

Importantly, distortions in MR images can be substantially reduced when selecting the appropriate settings at the MR scanner. However, the diagnostic radiologist, who frequently is the one who obtains the MR images used for treatment planning, may be virtually unaware of the problems associated with distortion-related imaging errors in radiotherapy planning as distortion is far less of a problem in diagnostic radiology. In the following section we therefore want to discuss the most important types of distortion in MR images and how they can be minimized.

Distortions most relevant to MRI-based intracranial radiotherapy are those due to nonlinearities of the gradient coils and those due to inhomogeneities in the main magnetic field (B0) [35]. Inhomogeneities in the B0 field occur due to residual imperfections of the main magnet but also because the patient himself disturbs the magnetic field due to magnetic susceptibility effects. These three most relevant types of distortion are usually grouped into system-related (gradient nonlinearity-related distortions and main magnet imperfections) and patient-induced (susceptibility effect-induced distortions) but a more practical approach is to differentiate between sequence-independent (gradient nonlinearity-related distortions) and sequence-dependent distortions (inhomogeneities of the static magnetic field due to magnet imperfections or due to patient-induced perturbations) [24, 31, 35].

### Sequence-independent gradient nonlinearity-related distortions

In MRI, three gradient coils superimpose magnetic field gradients onto the main magnetic field in the X, Y and Z dimensions. Linearly varying gradients of magnetic field strength are the basis for spatial encoding in MRI and subsequent image reconstruction [2, 30, 31, 35–37]. However, because of additional requirements on gradient coil design like the need for fast gradient switching times and to avoid nerve stimulation, gradient nonlinearities are present especially at the periphery of the scanner (Fig. 4; [37, 38]). These gradient nonlinearities lead to spatial distortions during image reconstruction increasing with radial distance from the isocenter [31, 32, 34, 38, 39]. Gradient nonlinearity-related distortions are usually the most significant type of distortion in MRI [30]. Gradient nonlinearities are specific to every MR scanner model, i.e. system-related and sequence-independent. Distortions due to gradient nonlinearities therefore do not change with different sequence settings but will differ when using different scanner models [2, 24, 30–32, 38]. However, image distortions due to gradient nonlinearities may change for the same scanner when the patient is positioned differently relative to the gradient fields [24, 32]. The amount of distortion to be expected from gradient nonlinearities depends on the scanner model and the patient position relative to the isocenter, and may reach several millimeters at the periphery of the brain ([32, 38]; Fig. 4g–h).

Fortunately, as gradient nonlinearity-related distortions are a constant property of the gradient coil set known to the manufacturer [37, 38], they can be corrected using vendor-specific distortion correction if correctly configured at the MR scanner.

Vendor-specific distortion correction is usually implemented via a postprocessing step using a deformable registration like wrapping of images that also requires resampling and intensity correction of images [32, 38, 40]. This may change some image and noise characteristics, which may be undesirable for diagnostic radiology [20].

It is therefore important to assure that the MR images obtained for cranial radiotherapy simulation are properly distortion corrected.

For correction of gradient nonlinearity-related distortions, 3D and 2D correction is frequently available with 3D correction commonly being considered the preferred setting for most radiotherapy treatment tasks as 2D correction does not correct for through-plane distortions (Fig. 4; [38, 39]). In fact, vendor-specific 3D distortion correction was regarded as a minimum requirement in a 2016 consensus paper on MRI simulation published in *Radiotherapy and Oncology* with additional corrections required based on on-site measurements [6]. Importantly, while vendor-specific 3D correction should be the minimum for radiotherapy planning, in our experience most MRI sequences acquired in external departments are only 2D corrected. In fact, some scanners do not apply distortion correction as routine postprocessing at all [30].

Seibert et al. assessed the clinical impact of gradient nonlinearity-related distortions in cranial radiosurgery comparing 3D corrected with uncorrected images. They found a median GTV displacement of 1.2 mm and a maximum GTV displacement of 3.9 mm in uncorrected images. As a result, geographic miss would have occurred in 8 of 28 lesions if uncorrected images had been used [5].

Nondistortion-corrected series may be marked in the series name (e.g. with an “_nd”) and information on distortion correction (i.e. 3D, 2D or none) is usually stored in the DICOM header albeit in different fields (e.g. at 0008 × 0008 “Image Type”, 0008 × 9206 “Volumetric Properties” and in private fields). Importantly, some residual distortion may remain after vendor-specific correction and properties of gradient fields may change over time. As stated therefore in the 2016 consensus paper by Paulson et al., residual distortion after vendor-specific correction should be characterized using phantoms and corrected if necessary [6]. Multiple authors have described rectifying residual gradient nonlinearity distortions using 3D deformation vector fields obtained via phantom measurements [30, 37, 41].

### Sequence-dependent distortions

Distortion correction via postprocessing configured at the scanner can however not correct distortions because of magnet imperfections or tissue susceptibility [2, 38]. Similar to nonlinearities of gradient coils, inhomogeneities of the main magnetic field (= B0 inhomogeneities) also lead to distortions in MR images. These B0 inhomogeneities arise due to imperfections in magnet design but also due magnetic perturbations induced by the patient himself [2]. In stark contrast to gradient nonlinearity-related distortions, distortions due to main magnetic field inhomogeneities change with different sequence settings. Also, while gradient nonlinearity-related displacements occur in all three dimensions, B0 inhomogeneity-related distortions in regular 3D-sequences only occur in the frequency-encoding dimension. As the slice selection process in 2D-sequences is also disturbed by B0 inhomogeneities, 2D-sequences are more susceptible to B0 inhomogeneity-related distortions than 3D-sequences [2, 30, 42]. The effect of main magnetic field inhomogeneities on image distortions increases with field strength, i.e. displacements increase at 3 T in comparison to 1.5 T in the absence of other compensating factors [2, 43].

Patient-induced perturbations of the main magnetic field occur because of differences in magnetic susceptibility, which is the physical property of material becoming magnetized inside an external magnetic field [2, 35, 44]. The greatest susceptibility differences and therefore the most severe distortions occur at air–bone interfaces [35, 44]. In the case of intracranial radiotherapy, the most severe susceptibility-related distortions are therefore to be expected near the paranasal sinuses and the mastoid cells [42]. In a 2013 study, Wang et al. measured susceptibility-induced distortions in T1-MPRAGE sequences of the brain to be <0.5 mm in 86.9% of the imaged volume (3 T; bandwidth 180 Hz/pixel; patient-specific automated shimming). However, while average displacement was low for the whole imaged volume, average distortions at sinus air–bone boundaries was 1.6 mm. While degrading with distance, these distortions extended into the adjacent brain and optic system and still measured 0.8 mm at a distance of 12 mm, which is clinically relevant for RT targets in parts of the brain adjacent to the sinuses and mastoid cells [42]. In addition, large susceptibility differences and consecutive distortions may also occur at the site of metallic implants like surgical clips [42].

While vendor-specific distortion correction does not correct for distortions due to B0 inhomogeneities, they can be ameliorated with increasing readout bandwidth, using 3D- instead of 2D-sequences [45] and activating patient-specific active shimming.

Increasing readout bandwidth, decreases all distortions due to B0 inhomogeneities in a reciprocal fashion [35, 42, 46, 47]. The downside, however, is that the signal-to-noise ratio (SNR) also has an inverse relationship to the square-root of the readout bandwidth [47]. Most radiology departments therefore favor lower bandwidths as this allows for reduced imaging time while maintaining a high signal-to-noise ratio. However, in stark contrast to the preferential settings for routine radiological imaging, MRI for radiotherapy planning requires higher bandwidths to reduce distortions from B0 inhomogeneities [13]. The 2016 Consensus report on MRI simulation in radiotherapy explicitly recommends increasing sequence readout bandwidths while accepting consecutive loss of SNR [6]. SNR loss due to higher bandwidth can be compensated for with strategies that increase SNR like increasing measurement time, optimized coil selection and decreased motion artifacts due to immobilization. As mentioned before, 3 T scanners would be expected to suffer more from B0 inhomogeneity-related distortions. However, the increasing field strength also increases SNR which enables higher compensatory readout bandwidths [30, 43, 46]. In addition, a modern 3 T scanner usually will have better shimming to reduce B0 inhomogeneities than most older 1.5 T scanners.

Active shimming can reduce system-related and patient-induced B0 inhomogeneities directly using a set of specialized shim-coils. B0 inhomogeneities are first measured using a patient-based but fast and low-resolution phase difference mapping technique and subsequently reduced with the shim coils [42, 48]. However, patient-specific active shimming needs to be available at the scanner and properly configured.

If patient-specific active shimming and RT-optimized bandwidth settings are not completely successful in reducing B0 inhomogeneity-related distortions to an acceptable level, additional correction is possible by obtaining an improved higher resolution phase difference map which can be used to correct patient-specific B0 distortion via image postprocessing [31, 49]. A reverse gradient method has also been proposed to correct B0 inhomogeneity-related distortions but requires obtaining every sequence two-fold and may lead to degraded image quality in most clinical settings [31, 36, 50, 51].

An additional source of patient-related distortions is the chemical shift, with the fat–water shift being the most prominent example. It causes adipose tissues like fat with a slightly different resonance frequency to be shifted along the frequency encoding direction. This effect is however also minimized by choosing higher bandwidths [52].

Current consensus defines that total distortions in MRI need to be less than 1 mm for stereotactic radiotherapy in the brain [6]. Enabling vendor-specific 3D distortion correction and patient-specific active shimming in the scanner software as well as selecting RT-optimized readout bandwidths before acquisition of the planning MRI are steps which are easy to implement and may sufficiently eliminate distortions in many cases.

It is however important to verify that the geometric accuracy required for brain stereotactic radiotherapy (<1 mm) is indeed achieved by measuring residual distortions. Given that wear and tear of individual components, wrong software settings or even small metallic objects like earrings/ear studs left inside the magnet may result in unnoticed distortions [6, 45], some form of regular quality assurance is mandatory to assure optimal images for brain stereotactic radiotherapy [6].

## Choosing optimal MRI sequences for radiotherapy planning

MRI for radiotherapy planning primarily needs to accurately depict the tumor perimeter in three-dimensional space for precise gross tumor volume (GTV) delineation.

**Fig. 5:**
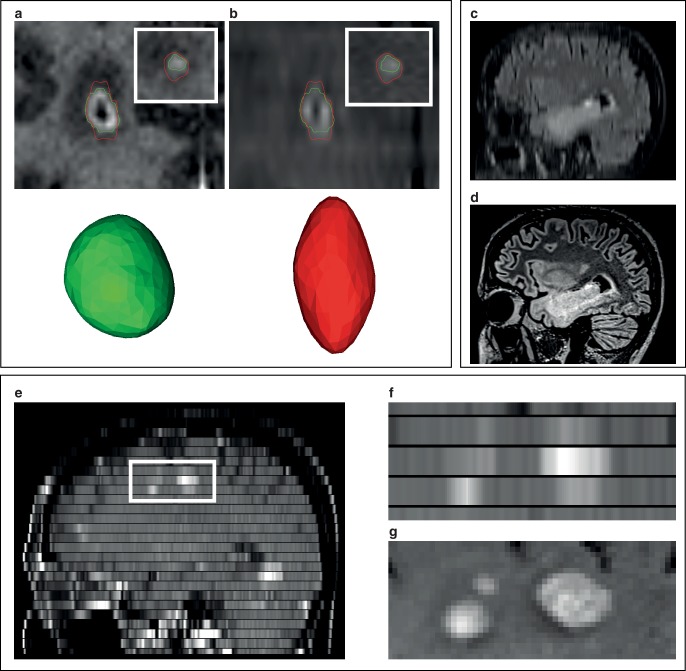
Advantage of high-resolution 3D-sequences. **a**, **b** Impact of slice thickness on GTV size: **a** 1 mm slice thickness; **b** 3 mm slice thickness. Coronal reconstruction with axial reconstruction (*inset*). *Bottom*: 3D rendering. GTV was defined based on the 1 mm (*green*) and 3 mm (*red*) dataset. *Note*: GTV size is substantially overestimated with 3 mm thick slices in the direction of lowest image resolution due to partial volume effects. **c**, **d** 3D T2-SPACE FLAIR (1 mm slice thickness—**d**) vs. conventional 2D T2-FLAIR (5 mm slice thickness—**c**) in a patient with glioma. **e** Visualization of slice gaps present in a routine 2D T1-TSE sequence. Slice gaps of 0.5 mm are present between 5 mm slices, which are not evident with routine inspection of images but could impair accurate tumor delineation and registration. **f** Enlarged view. **g** The 1 mm^3^ 3D-TSE (T1-SPACE) without gaps for comparison. Interpolation was inactivated to adequately illustrate gaps between slices

Isotropic 3D-sequences are usually best-suited for this task as they enable accurate multiplanar reconstruction and minimize over- or underestimation of GTV volume due to partial volume effects (Fig. 5; [53, 54]). In addition 3D-sequences are also less susceptible to B0 inhomogeneity-related distortions than 2D-sequences and image the brain continuously without gaps.

It has been shown that the volumetric error will exceed 10% if the GTV is visualized on less than 5 slices, which is particularly relevant for small brain metastases [54]. Partial volume effects usually lead to overestimation of the GTV volume if slice thickness is too high. This is also important when fusing multiple MRI series acquired in different planes (e.g. sagittal or coronal plane) as partial volume effects may accumulate and lead to imprecise contouring of the GTV. In addition, thick slices and image gaps could also lead to underestimation of tumor growth perpendicular to the imaging plane or miss small metastatic lesions (Fig. 5e–g).

### T1-MPRAGE (T1 3D-IR-GRE) vs. T1-SPACE (T1 3D-TSE) for delineation of brain metastases

**Fig. 6:**
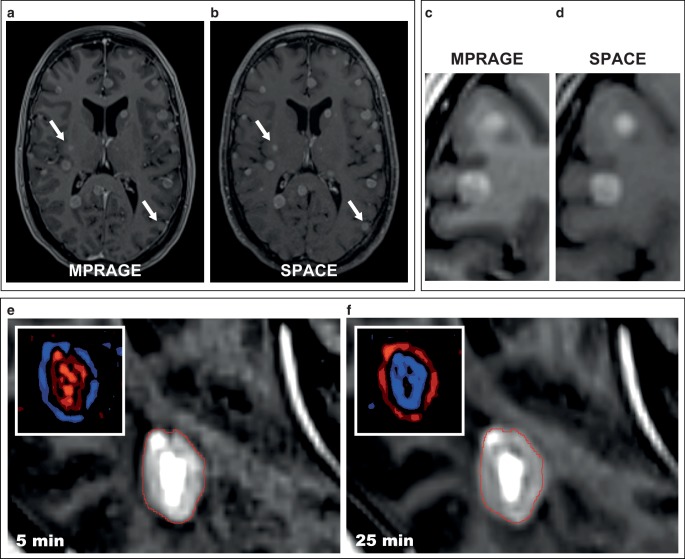
Sequence type and contrast-related parameters influence GTV discrimination. **a**, **b** T1-SPACE 3D-TSE sequence (**b**) vs. T1-MPRAGE IR-GE sequence (**a**). Some metastases are only very faintly visible in the T1-MPRAGE (*arrows*). *Note also*: Suppression of vessels and less contrast between gray and white matter in the T1-SPACE. **c**, **d** Close-up view. **e**, **f** Impact of the time interval between contrast administration and imaging. A brain metastasis appears significantly larger 25 min (**f**) vs. 5 min (**e**) after contrast administration. Inset: Difference map between early and late acquisition and vice versa. Higher signal intensity is visualized in *red*, lower in *blue*

Inversion-recovery gradient echo sequences (IR-GRE) like the T1-MPRAGE [55] have been the most commonly used 3D MR imaging technique for brain tumors and have been included in the standardized brain tumor imaging protocol (BTIP) [56, 57]. However, multiple sources suggest that a 3D-turbo-spin-echo (TSE) T1-SPACE could be superior to the frequently used T1-MPRAGE gradient-echo sequence for intracranial radiotherapy target volume delineation [56, 58–60]. While T1-SPACE provides less contrast between grey and white matter [56], this is negligible in most cases for radiotherapy treatment planning and may in fact even help with the delineation of intracranial metastases, as does the suppression of vessels in the T1-SPACE [60]. Conversely, T1-MPRAGE suffers from a known reduced enhancement if low contrast agent uptake is present, which could lead to underestimation of lesion boundaries (Fig. 6; [56, 61]).

In a very recent paper by Danieli et al. published in the *American Journal of Neuroradiology*, the authors systematically compared T1-MPRAGE, T1-SPACE and T1-VIBE in 16 brain metastases and 38 gliomas [56]. Importantly, they found the highest contrast rate (i.e. the difference in signal intensity between tumor and surrounding tissue) and the highest contrast-to-noise ratio for T1-SPACE. In a joint qualitative evaluation by a neuroradiologist and a neurosurgeon the T1-SPACE also achieved the best rating for visual conspicuity in all cases, whereas the T1-MPRAGE and T1-VIBE achieved the best rating only in 27.8% and 44.4% of lesions, respectively. Additionally, GTV volumes were largest when defined on the T1-SPACE (median 1.78 cm^3^) as compared to the T1-MPRAGE (1.36 cm^3^) and T1-VIBE (1.62 cm^3^). GTV definitions based on the T1-MPRAGE missed a median contrast-enhancing volume of 0.27 cm^3^ visualized in the T1-SPACE in 19.9% of cases, whereas only a median contrast-enhancing volume of 0.10 cm^3^ visualized in the T1-MPRAGE was missed in 7.4% of cases by the T1-SPACE in the reciprocal comparison. Interestingly, 15.8% of all brain metastases were missed on the T1-MPRAGE, whereas all lesions were visualized on the T1-SPACE 3D-TSE sequence. Importantly, the authors addressed the potential impact of different contrast phases by randomizing the order of sequences in each patient and by accounting for the order of acquisition in their analysis [56]. Furthermore, spin-echo sequences like the T1-SPACE come with the additional advantage of reduced metal artifacts in comparison to the gradient-echo based T1-MPRAGE, which is helpful when imaging brain tumor patients with shunts or surgical clips [20, 35].

## Optimizing contrast agent administration

The dose of gadolinium-based contrast agent (GBCA) and the time interval between contrast application and measurement are additional important parameters that may affect the delineations of lesions in T1-based MR sequences (Fig. 6e–f; [53, 62–64]). Yuh et al. compared early (10 min) and late imaging (20 min) after standard dose gadoteridol [62]. In metastases <5 mm, 40.6% of lesions were visualized after 10 min, whereas 75.0% could be seen after 20 min. The remaining lesions were only visualized after an additional bolus of double-dose gadoteridol, indicating the value of increased doses of GBCA [62]. Kushnisky et al. reported similar findings and found more brain metastases at 15 min after GBCA administration as compared to 5 min. Importantly, they also found an increase in metastasis volume with imaging at 10 vs. 5 min and also at 15 vs. 10 min post-contrast [64]. Balériaux et al. found an increasing number of metastases and improved conspicuity of lesions with increasing cumulative dose after multiple sequential injections of gadobenate dimeglumine [63]. Double-dose contrast agent in our experience is especially helpful in difficult to visualize lesions with low contrast-uptake [53]. Finally, the type of GBCA also has a known effect on brain metastases conspicuity. For example in a 2013 literature review by Anzalone et al., gadobutrol (Gadovist) was superior to other GBCAs in terms of the number of metastases detected and improved lesion visualization [53].

## Summary

MR imaging for radiotherapy treatment planning is an integral part of the radiotherapy planning process whose critical nature for precise treatment delivery is easily overlooked. In the past, MRI and radiotherapy have largely evolved independently from each other with MRI having been optimized primarily for the requirements of diagnostic radiology. The recent advent of MRI-LINACs has put MRI into the main focus of radiation oncology. Improved MRI simulation has a significant potential for improving clinical results in brain stereotactic radiotherapy. At the same time the potential for clinically relevant errors is substantial when using MR images that have not been optimized for the specific requirements of stereotactic radiotherapy.

It is important to assure that suitable MR images are used for treatment planning. Requirements on MR imaging for radiotherapy differ from routine diagnostic imaging and need to be discussed with the radiologist, from whom MR images are obtained. The most important topics that need to be addressed when using MRI in brain stereotactic radiotherapy include the acquisition of distortion-free images, the minimization of the time interval between imaging and treatment delivery and the use of RT-optimized 3D-sequences.
